# Demographic structure, consumption composition, and household health investment: evidence from sports and fitness expenditure

**DOI:** 10.3389/fpubh.2026.1815361

**Published:** 2026-09-23

**Authors:** Ziyue Wang, Haoran Bai

**Affiliations:** 1 School of Physical Education and Sport Science, Fujian Normal University, Fuzhou, China; 2 Advanced Institute of Finance, Henan University, Zhengzhou, China

**Keywords:** demographic structure, dependency ratio, health/fitness expenditure, health-related expenditure, household consumption

## Abstract

**Purpose:**

This study examines how household age structure is associated with health-related expenditure in China, distinguishing broad medical and healthcare expenditure from narrower health/fitness expenditure.

**Methods:**

Using nationally representative China Household Finance Survey data from 2015, 2017, 2019, and 2021, we estimate associations between old-age and child dependency ratios and two expenditure outcomes and examine potential mechanisms through precautionary saving and household disposable income.

**Results:**

Old-age dependency is positively and relatively consistently associated with both broad medical and healthcare expenditure and narrow health/fitness expenditure. The association for child dependency is less stable across outcomes and specifications, but the mediation results are consistent with the possibility that household income constraints are relevant for discretionary health/fitness expenditure. Robustness, heterogeneity, and supplementary instrumental-variable analyses support an associational interpretation rather than a strong causal claim.

**Conclusion:**

Demographic structure is linked not only to the level of household health-related expenditure but also to its composition. Distinguishing old-age and child dependency helps clarify how demographic transition may shape household capacity for preventive and discretionary health investment.

## 1 Introduction

Population ageing and declining fertility have become defining features of contemporary demographic transition, with important implications for household behavior and health outcomes (1). A growing body of literature suggests that changes in age structure influence expenditure patterns through both demand-side and resource-allocation channels, particularly within the life-cycle framework and in the domain of health-related expenditure (2). However, existing research still provides only a partial account of how demographic structure shapes household health-related expenditure.

Much of the health-expenditure literature focuses on medical or out-of-pocket healthcare spending and the associated financial burden (3), with less attention to more discretionary health-promoting expenditure. This emphasis may obscure important differences in the underlying mechanisms, because essential healthcare expenditure is largely driven by health needs, whereas spending on health- and fitness-related activities may be more sensitive to socioeconomic conditions, time availability, and household resource constraints. As a result, it remains unclear whether demographic change is associated with expansion or contraction in health-related expenditure overall.

A further limitation is that many studies focus on a single dimension of demographic change, usually population ageing, without jointly distinguishing between the old-age dependency ratio and the child dependency ratio. Yet these two forms of dependency capture fundamentally different sources of household pressure. Population ageing is more closely linked to healthcare demand, health shocks, and long-term-care expenditure (4, 5), whereas child dependency is more closely linked to resource dilution and competing investment needs. Failing to distinguish between them may therefore blur the relationship between demographic structure and expenditure allocation.

This study addresses these gaps by examining how household age structure is associated with health-related expenditure, with particular attention to health/fitness expenditure. Using nationally representative data from the China Household Finance Survey (CHFS), we distinguish between broad medical and healthcare expenditure and narrow health/fitness expenditure, thereby separating more necessity-driven spending from more discretionary components of household expenditure. We then examine the old-age dependency ratio and the child dependency ratio within a unified analytical framework and explore two potential channels: precautionary saving and household disposable income per capita.

Rather than presenting demographic structure as a single household characteristic, the empirical analysis asks whether old-age and child dependency are linked to health-related expenditure through different pathways. This framing is important for public health because demographic pressure may affect not only realized medical spending, but also households’ capacity to sustain preventive and health-promoting expenditure such as sports and fitness consumption.

This study contributes to the literature in three ways. First, it distinguishes broad medical and healthcare expenditure from narrower health/fitness expenditure, thereby separating necessity-driven spending from more discretionary health-promoting consumption. Second, it analyzes old-age and child dependency within a unified framework while allowing them to operate through different mechanisms. Third, it links household demographic structure to public-health concerns about unequal opportunities for preventive health investment before illness occurs.

### 1.1 Demographic structure and household health-related expenditure

A substantial body of literature in household economics emphasizes that consumption is closely linked to demographic structure, particularly within the life-cycle framework. According to the life-cycle hypothesis, individuals smooth consumption over time based on expected lifetime income, while demographic factors such as age composition and dependency ratios shape both consumption needs and resource constraints. At the household level, dependency ratios capture the relative burden of non-working members on working-age individuals and, consequently, influence both the allocation of income and the composition of expenditure. Related evidence shows that medical-expense risk can shape saving in later life (6), while consumption inequality reflects differences in household resources and consumption opportunities over the life cycle (7).

Existing studies suggest that demographic structure affects consumption through two broad channels. First, changes in population composition directly affect demand for specific goods and services, such as healthcare, education, and housing. This demand-side channel is particularly evident in ageing societies, where demographic shifts lead to increased demand for health care and long-term care services (8). Second, demographic pressures influence the availability and allocation of household resources, thereby affecting discretionary consumption through budget constraints and intra-household decision-making. This resource-constraint channel is especially relevant for sports and physical activity, as recent evidence shows that participation in physical activity and related behaviors is strongly influenced by socioeconomic inequalities, time availability, and financial constraints (9, 10). However, the relative importance of these channels may vary across demographic groups and consumption categories, leading to heterogeneous empirical findings.

### 1.2 Population ageing and health-related expenditures

Ageing is associated with greater demand for medical and long-term-care services and with rising healthcare expenditure (2, 5, 8, 11). It may also increase preventive and health-management activity as older adults engage in physical activity and other healthy-ageing behaviors (12–15).

Ageing may also heighten uncertainty about future health costs and create a precautionary-saving motive, potentially crowding out discretionary consumption (6, 16). The importance of this offsetting channel therefore remains uncertain.

Based on the above discussion, old-age dependency may affect household health-related expenditure through both healthcare demand and precautionary motives, although the demand-side mechanism is expected to dominate. Specifically, because broad medical and healthcare expenditure mainly reflects healthcare needs and service utilization, old-age dependency is expected to show a relatively consistent positive association with this expenditure category. In contrast, narrow health/fitness expenditure represents a broader form of health investment and discretionary consumption, for which the positive effect of active ageing may coexist with the potential crowding-out effect of precautionary saving. Therefore, the overall association between old-age dependency and different types of health-related expenditure remains an empirical question. Accordingly, this study proposes the following hypotheses:

*H1*: A higher old-age dependency ratio is expected to be positively associated with household health-related expenditure.

*H2*: If the old-age dependency ratio is positively associated with household health-related expenditure, the pattern should be more consistent with health-related expenditure demand than with crowding-out through precautionary saving.

### 1.3 Child dependency, resource allocation, and health/fitness expenditure

Organized activities, including sports, can contribute to children’s health and development (17). This provides a basis for parental investment motives, although participation itself is also shaped by household economic constraints (18).

Yet a higher child dependency ratio also dilutes per capita resources and raises essential spending, tightening budgets for discretionary sports and fitness expenditure (19, 20).

These forces may affect expenditure categories differently: essential health or education spending may be maintained, while discretionary sports and fitness spending is more vulnerable to financial, access, time, and parental-resource constraints (21–23).

Based on the above discussion, child dependency may generate both investment motives and resource constraints, and these forces may operate differently across expenditure categories. Unlike old-age dependency, which is primarily associated with healthcare demand, child dependency reflects a combination of investment needs and household resource constraints. As a result, child dependency may increase essential health-related spending through parental investment motives while simultaneously reducing discretionary health-promoting expenditure when household budgets become constrained. These competing mechanisms suggest that the association between child dependency and household health-related expenditure may vary depending on the expenditure category. Accordingly, this study proposes the following hypothesis:

*H3*: The association between the child dependency ratio and household health-related expenditure may differ across expenditure types because investment motives and resource constraints can operate simultaneously.

### 1.4 Sports consumption, socioeconomic inequality, and heterogeneity

Because sports and fitness consumption requires both money and time and is usually more discretionary than medical expenditure, it is especially sensitive to household income, parental resources, and access to facilities (24).

Accordingly, participation is systematically higher among households with greater socioeconomic resources, and such disparities can persist or widen over time (25).

These disparities make health/fitness expenditure a useful lens for examining how demographic pressure interacts with household resources. Compared with essential medical expenditure, discretionary health-promoting expenditure is more sensitive to household budget constraints because it requires additional financial resources and consumption flexibility. If child dependency tightens the household budget, discretionary health-promoting expenditure should therefore be more affected than essential medical expenditure. More generally, evidence that income risk can induce precautionary saving is consistent with reduced flexibility in current discretionary consumption (26). This resource-constraint perspective further suggests that household disposable income may represent an important pathway linking child dependency to health/fitness expenditure. Accordingly, this study proposes the following hypothesis:

*H4*: Any association between the child dependency ratio and narrower health/fitness expenditure may partly operate through household per capita disposable income.

The child-dependency association may also vary with socioeconomic context. Higher-resource households may have more discretionary margins to adjust, while lower-resource households may face binding constraints; urban–rural differences in access may further shape observed spending.

These considerations make heterogeneity an empirical question and motivate the following hypothesis:

*H5*: The association between the child dependency ratio and household health-related expenditure is likely to vary across urban-rural and income groups because households differ in consumption flexibility and access to health-promoting activities.

## 2 Materials and methods

### 2.1 Data sources and sample construction

The China Household Finance Survey (CHFS), conducted by the Survey and Research Center for China Household Finance, is a nationally representative household survey covering finances, consumption, demographic characteristics, employment, and social protection.

In this study, we use four waves of CHFS data from 2015, 2017, 2019, and 2021. Pooling these waves yields 133,970 household-year observations before applying the common-sample restrictions used in the baseline models. The main analysis is based on the harmonized 2015–2021 sample, while the 2013 wave is used only in the extended robustness test because several controls cannot be defined consistently across all years.

Baseline regressions use complete cases because some controls and mediators are missing. Continuous variables are winsorized at the 1st and 99th percentiles. Although the CHFS is not designed specifically for sports and fitness spending, it provides nationally representative household data suitable for comparing demographic structure and expenditure composition.

### 2.2 Outcome variables

We define two outcome variables to separate broad necessity-oriented spending from narrower health-promoting consumption.

Broad medical and healthcare expenditure is constructed using ‘medical and health care expenditure in the past year’ in 2015 and total household medical and healthcare expenditure in 2017–2021. The harmonized measure is transformed as ln(1 + x) to accommodate skewness and zeros; it primarily captures medical and general healthcare spending. This harmonization ensures comparability across survey waves despite differences in questionnaire design. Conceptually, this category captures both essential medical spending and general health-related expenditures, which are treated here as more necessity-oriented and more closely related to underlying health conditions and medical needs. As such, it primarily reflects demand-driven responses to health shocks and aging-related care needs.

Narrow health/fitness expenditure is constructed from 2017 to 2021 items on health maintenance and fitness activities and excludes core medical treatment. It is also transformed as ln(1 + x). It captures spending related to health maintenance and physical activity, behaviors that are central to health promotion (27); relative to core medical treatment, it is treated here as the narrower and more discretionary expenditure outcome. In contrast to the broad measure, this category isolates health-promoting consumption, which is more discretionary and reflects households’ voluntary investment in health and well-being. Consequently, it better captures choice-based consumption behavior, which is more sensitive to income constraints, preferences, and intertemporal allocation decisions.

This dual-outcome specification offers two advantages. First, it avoids conflating necessity-driven medical expenditure with discretionary health-promoting expenditure, thereby reducing measurement ambiguity in the dependent variable. Second, it allows a clearer assessment of the different channels through which demographic structure may be associated with household behavior. In particular, increases in the old-age dependency ratio may be associated with higher demand for medical care, whereas changes in the child dependency ratio may be more closely related to budget constraints and expenditure trade-offs. Distinguishing between these two outcomes therefore helps clarify the mechanisms underlying household health-related expenditure decisions.

### 2.3 Core explanatory variables

The old-age dependency ratio is the number of household members aged 65 and above divided by the number aged 15–64; the child dependency ratio is the number aged 0–14 divided by the number aged 15–64. Both are calculated from individual-level ages within each household, following the standard age-group definitions used by the United Nations (28).

The two ratios are entered in separate regressions because they represent different demographic components and joint inclusion may create collinearity.

### 2.4 Mediating variables

Mediation analyses pair old-age dependency with a liquid-assets proxy for precautionary saving and child dependency with disposable income per capita; they assess patterns consistent with potential pathways, not causal mediation. This design is motivated by the premise that old-age and child dependency capture fundamentally different sources of household pressure and therefore should not be assumed to operate through a common mechanism.

#### 2.4.1 Precautionary savings channel

For the old-age dependency ratio, we examine precautionary saving as a potential mediating channel. From the perspective of household economics, an increase in the old-age dependency ratio may raise uncertainty regarding future health-related expenditures and long-term care needs. In response, households may increase precautionary saving, which could reduce resources available for more discretionary forms of spending. As the CHFS does not provide a direct measure of precautionary saving, we construct a proxy based on household liquid asset holdings. Precautionary saving is proxied by household liquid assets, defined as demand deposits plus cash and transformed as ln(1 + x). This operational measure represents readily available financial buffers that households could draw on against future health and care costs; its interpretation is motivated by evidence on medical-expense risk and saving behavior (6, 16) and on substantial out-of-pocket home-care costs among older adults (29).

#### 2.4.2 Income channel

The child-dependency mediator is household disposable income per capita. It captures the resources available per household member and is therefore relevant to budget-sensitive health/fitness expenditure. According to life-cycle theory and household resource-allocation theory, changes in dependency structure may be associated with both income-generating capacity and expenditure needs. Specifically, a higher child dependency ratio may place greater financial pressure on working-age household members because a larger share of resources may need to be allocated to dependent children. This pattern may be associated with lower per capita disposable income and tighter household budget constraints. In addition, increased child-related expenditures, such as education and childcare, may further shift household resources away from discretionary categories. Consistent with evidence linking family socioeconomic conditions to children’s physical activity and sports participation (30), this test asks whether estimates are compatible with an income-constraint pathway; it does not establish causal mediation.

### 2.5 Control variables

Household-head controls include age, gender, marital status, health status, education, and working time (average working days per month). Household controls are log total assets, log total consumption, and household size. An indicator for rural residence (rural = 1) and year fixed effects account for location and common time-specific factors.

### 2.6 Conceptual framework

Figure 1 summarizes the empirical logic of the study. The framework treats household age structure as the exposure, distinguishes broad medical and healthcare expenditure from narrow health/fitness expenditure as the two outcome variables, and maps old-age and child dependency onto distinct hypothesized channels. The figure also indicates how the main empirical components—baseline regressions, mediation analysis, heterogeneity analysis, and supplementary instrumental-variable diagnostics—are organized around the core exposure-potential pathway-outcome structure.

**Figure 1:**
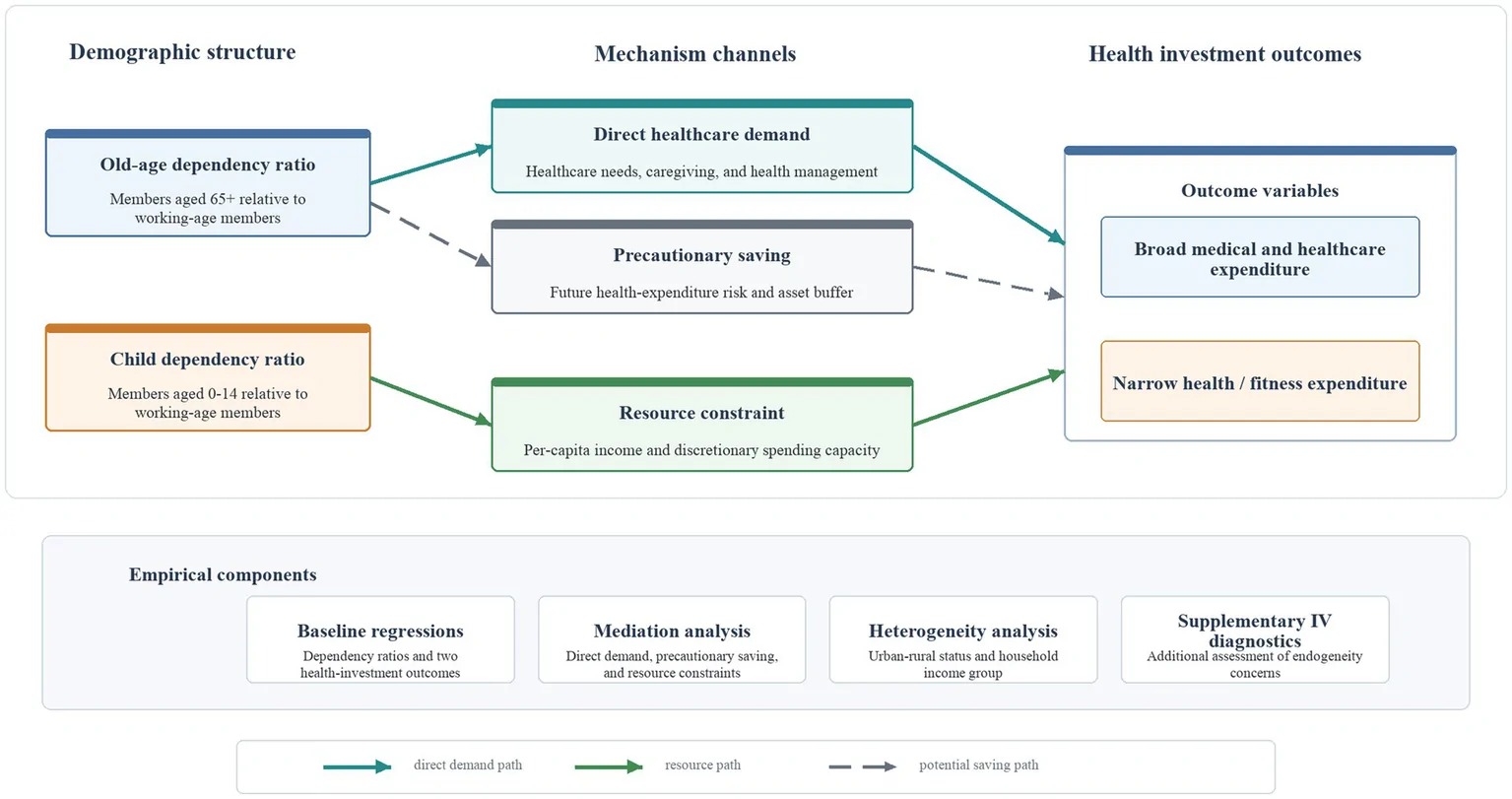
Conceptual framework and empirical components.

### 2.7 Empirical strategy

To estimate the association between demographic structure and household health-related expenditure, we pool the CHFS waves and estimate weighted regressions with year fixed effects. Because the empirical sample is a pooled repeated cross-section rather than a balanced household panel, the main specification is a pooled OLS model with year fixed effects and standard errors clustered at the household level. In addition, a substantial share of households is observed in only one wave, and household demographic structure changes only gradually over time, so a household fixed-effects specification would substantially reduce the effective sample and rely on limited within-household variation.

The baseline specification is:


$$Y_{\mathrm{it}}=\alpha +\mathrm{\beta De}p_{\mathrm{it}}+\gamma^{\prime }X_{\mathrm{it}}+\delta_{t}+\varepsilon_{\mathrm{it}}$$ (1)

In Equation 1, $Y_{\mathrm{it}}$ denotes either broad medical and health care expenditure or narrow health/fitness expenditure for household i in year t . $\mathrm{De}p_{\mathrm{it}}$ denotes either the old-age dependency ratio or the child dependency ratio, estimated in separate regressions. $X_{\mathrm{it}}$ is the vector of control variables, and $\delta_{t}$ captures year fixed effects. $\varepsilon_{\mathrm{it}}$ is the error term.

To examine the proposed channels, we estimate the following mediation equations:


$$M_{\mathrm{it}}=\alpha_{m}+\mathrm{\theta De}p_{\mathrm{it}}+\gamma_{m}^{\prime }X_{\mathrm{it}}+\delta_{t}+u_{\mathrm{it}}$$ (2)

$$Y_{\mathrm{it}}=\alpha_{y}+\beta^{\prime }\mathrm{De}p_{\mathrm{it}}+\lambda M_{\mathrm{it}}+\gamma_{y}^{\prime }X_{\mathrm{it}}+\delta_{t}+v_{\mathrm{it}}$$ (3)

In Equations 2, 3, $M_{\mathrm{it}}$ denotes the mediator. For old-age dependency, the mediator is precautionary saving proxied by liquid asset holdings; for child dependency, the mediator is household disposable income per capita. This strategy is intended to provide evidence on plausible transmission channels rather than definitive causal mediation.

## 3 Results

### 3.1 Sample and descriptive characteristics

The pooled CHFS sample from 2015, 2017, 2019, and 2021 contains 133,970 household-year observations before applying the common-sample restrictions. After restricting the data to observations with non-missing outcome variables and complete covariates, the baseline broad medical and healthcare expenditure regressions use 47,749 household-year observations, while the narrow health/fitness expenditure regressions use 35,482 observations. The 2013 wave is used only in the extended robustness check because several controls cannot be defined consistently across all years.

Descriptive statistics for the main variables are reported in Supplementary Table S1. The mean (median) log value of broad medical and healthcare expenditure is 6.890 (7.758), indicating that this expenditure category is widespread in the pooled sample. By contrast, the mean log value of narrow health/fitness expenditure is only 0.968, and both the median and interquartile range are zero, indicating that this type of health-promoting expenditure is much less common at the household level.

For the core explanatory variables, the mean values of the old-age dependency ratio and the child dependency ratio are 0.205 and 0.204, respectively, although the former exhibits greater dispersion. Among the mediating variables, the mean log value of the precautionary-saving proxy is 7.637, whereas that of household disposable income per capita is 9.188. The control variables display patterns consistent with typical household survey data: the average age of the household head is 55.18 years, 75.36% are male, 84.99% are married, and rural households account for 33.85% of the sample. Overall, the sample exhibits substantial economic and demographic heterogeneity, providing a sound basis for the subsequent analyses.

### 3.2 Benchmark regression results

Table 1 reports the baseline regression results. These coefficient patterns are also illustrated in Figure 2. The old-age dependency ratio is positively and statistically significantly associated with both broad medical and healthcare expenditure (coefficient = 0.9901, *p* < 0.001) and narrow health/fitness expenditure (coefficient = 0.3834, *p* < 0.001). By contrast, the child dependency ratio is positively associated with broad medical and healthcare expenditure (coefficient = 0.1530, *p* = 0.0484) but is not significantly associated with narrow health/fitness expenditure (coefficient = −0.1081, *p* = 0.1631). Because both dependent variables are measured as ln(1 + x), the coefficients can be approximately interpreted as percentage differences in expenditure outcomes. For example, a 0.10 increase in the old-age dependency ratio is associated with approximately 10.4 and 3.9% higher levels of broad medical and healthcare expenditure and narrow health/fitness expenditure, respectively, holding other covariates constant. These baseline estimates are associational and do not establish causal effects.

**Table 1:** Baseline regression results.

Variable	(1)	(2)	(3)	(4)
Outcome	Broad med./Health exp.	Broad med./Health exp.	Narrow health/Fitness exp.	Narrow health/Fitness exp.
Old-age dep. ratio	0.9901*** (0.0627)		0.3834*** (0.0834)	
Child dep. Ratio		0.1530** (0.0775)		−0.1081 (0.0775)
Controls	Yes	Yes	Yes	Yes
Year fixed effects	Yes	Yes	Yes	Yes
*N*	47,749	47,749	35,482	35,482
R-squared	0.1252	0.1171	0.1093	0.1073

Household-level clustered standard errors are reported in parentheses. All regressions control for household and household-head characteristics and include year fixed effects. *, **, and *** denote significance at the 10, 5, and 1% levels, respectively.

**Figure 2:**
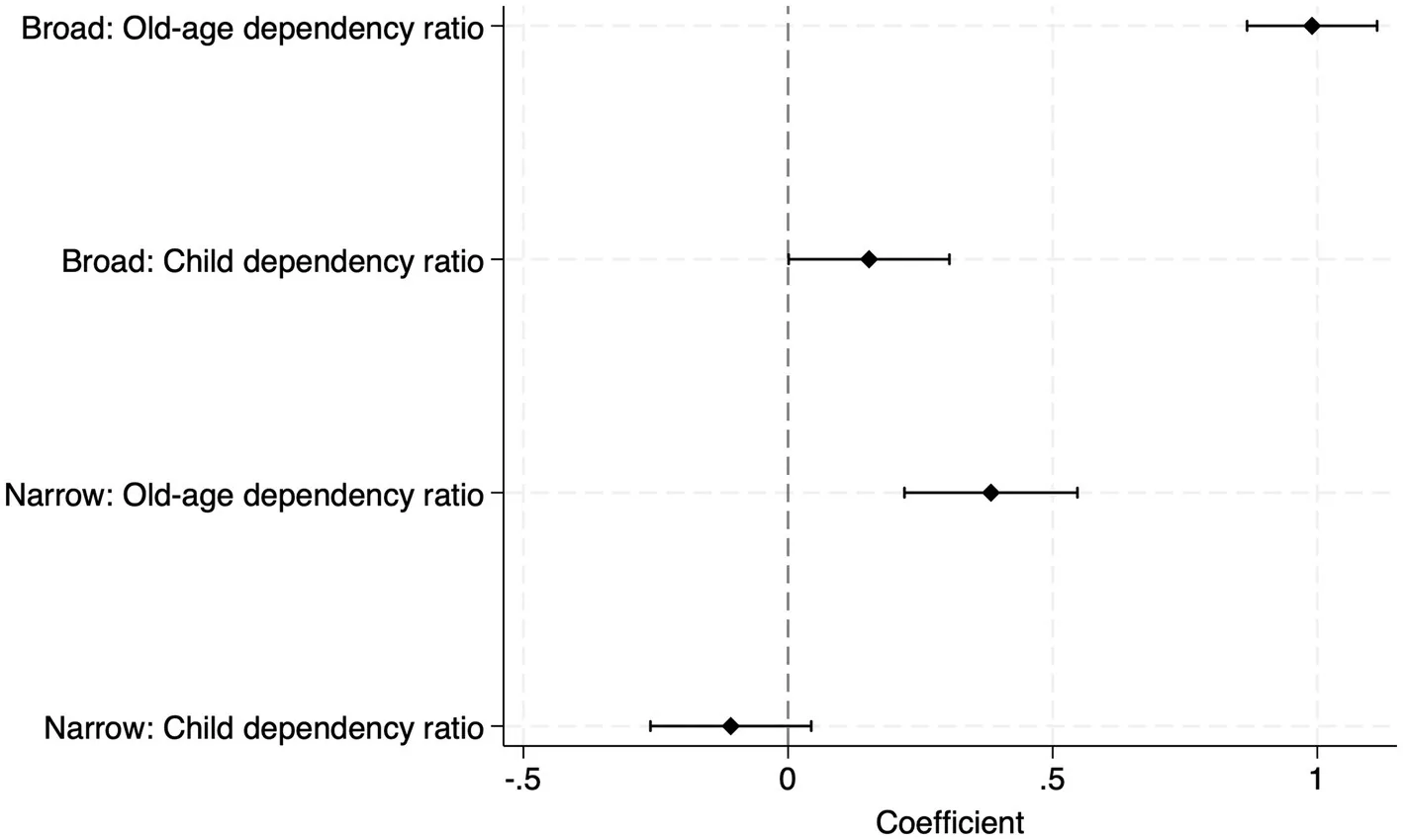
Baseline regression coefficient estimates. Points report coefficient estimates and horizontal lines denote 95% confidence intervals.

### 3.3 Mediation analysis

Table 2 reports the mediation results for the two proposed channels. For the old-age dependency ratio, the indirect effect through precautionary saving is statistically insignificant in both the broad expenditure specification (indirect effect = −0.0040, *p* = 0.3088) and the narrow health/fitness specification (indirect effect = −0.0037, *p* = 0.3920). This pattern suggests that the positive the positive association of old-age dependency association is more consistent with direct expenditure demand than with a savings-mediated reduction in current consumption.

**Table 2:** Mediation analysis.

Path/Statistic	(1)	(2)	(3)	(4)
Path/Outcome	Old-age dependency saving → broad	Child → income → broad	Old-age dependency saving → narrow	Child → income → narrow
A-path coefficient	−0.1019 (0.0985)	−0.3326*** (0.0360)	−0.0961 (0.1113)	−0.3512*** (0.0410)
B-path coefficient	0.0395*** (0.0069)	0.0575*** (0.0202)	0.0381*** (0.0058)	0.0546*** (0.0147)
Direct effect	0.9941*** (0.0627)	0.1721** (0.0772)	0.3870*** (0.0828)	−0.0889 (0.0780)
Indirect effect	−0.0040 (0.0040)	−0.0191*** (0.0070)	−0.0037 (0.0043)	−0.0192*** (0.0056)
Controls	Yes	Yes	Yes	Yes
Year fixed effects	Yes	Yes	Yes	Yes
*N*	47,749	47,749	35,482	35,482

Household-level clustered standard errors are reported in parentheses. All regressions control for household and household-head characteristics and include year fixed effects. *, **, and *** denote significance at the 10, 5, and 1% levels, respectively.

For the child dependency ratio, by contrast, the indirect effect through household disposable income per capita is statistically significant in both the broad expenditure specification (indirect effect = −0.0191, *p* = 0.0066) and the narrow health/fitness specification (indirect effect = −0.0192, *p* = 0.0007). The direct association remains positive and statistically significant in the broad specification (coefficient = 0.1721, *p* = 0.0258) but is negative and statistically insignificant in the narrow specification (coefficient = −0.0889, *p* = 0.2544). These mediation estimates identify patterns consistent with potential pathways, not causal transmission.

### 3.4 Robustness checks

Table 3 reports robustness checks using alternative estimators, samples, and time horizons.

**Table 3:** Robustness checks.

Variable	(1)	(2)	(3)	(4)	(5)	(6)	(7)	(8)
Outcome	PPML broad	PPML broad	PPML narrow	PPML narrow	Broad	Broad	Broad	Broad
Sample	2015–2021	2015–2021	2017–2021	2017–2021	2017–2021	2017–2021	2013–2021	2013–2021
Old-age dep. Ratio	0.4515*** (0.0452)		0.7083*** (0.1262)		1.0116*** (0.0685)		0.6131*** (0.0339)	
Child dep. Ratio		−0.0922 (0.0574)		−0.1741 (0.1329)		0.1192 (0.0873)		0.1389*** (0.0508)
Controls	Yes	Yes	Yes	Yes	Yes	Yes	Yes	Yes
Year fixed effects	Yes	Yes	Yes	Yes	Yes	Yes	Yes	Yes
Estimator	PPML	PPML	PPML	PPML	OLS	OLS	OLS	OLS
*N*	47,749	47,749	35,482	35,482	35,868	35,868	109,555	109,555
*R*-squared/pseudo *R*-squared					0.1374	0.1281	0.0952	0.0899

Household-level clustered standard errors are reported in parentheses. All regressions control for household and household-head characteristics and include year fixed effects. *, **, and *** denote significance at the 10, 5, and 1% levels, respectively.

Under PPML, the coefficient for old-age dependency remains positive and statistically significant for both outcomes, whereas the child-dependency coefficient is statistically insignificant in both specifications. Thus, the main pattern for old-age dependency pattern is not driven by the log transformation.

Using the harmonized 2017–2021 sample for broad expenditure, the coefficient for old-age dependency remains positive and statistically significant, while the child-dependency coefficient becomes statistically insignificant. In the reduced-control 2013–2021 specification, both coefficients are positive and statistically significant.

Across checks, old-age dependency is comparatively stable; child dependency varies with the sample window and specification and therefore warrants greater caution.

### 3.5 Heterogeneity analysis

Table 4 reports heterogeneity by urban–rural status and income; Figure 3 displays the urban–rural coefficient patterns.

**Table 4:** Heterogeneity analysis for broad medical and healthcare expenditure.

Variable	(1)	(2)	(3)	(4)	(5)	(6)	(7)	(8)
Group/Outcome	Urban	Rural	High income	Low income	Urban	Rural	High income	Low income
Dependent variable	Broad med./Health exp.	Broad med./Health exp.	Broad med./Health exp.	Broad med./Health exp.	Broad med./Health exp.	Broad med./Health exp.	Broad med./Health exp.	Broad med./Health exp.
Old-age dep. Ratio	1.1065*** (0.0804)	0.8547*** (0.0974)	1.1318*** (0.0982)	0.9257*** (0.0801)				
Child dep. Ratio					0.1868** (0.0952)	0.0442 (0.1344)	0.2294* (0.1318)	0.0822 (0.0953)
Controls	Yes	Yes	Yes	Yes	Yes	Yes	Yes	Yes
Year fixed effects	Yes	Yes	Yes	Yes	Yes	Yes	Yes	Yes
*N*	34,935	12,814	23,896	23,853	34,935	12,814	23,896	23,853
*R*-squared	0.1256	0.1300	0.1243	0.1316	0.1179	0.1205	0.1170	0.1223

Household-level clustered standard errors are reported in parentheses. All regressions control for household and household-head characteristics and include year fixed effects. *, **, and *** denote significance at the 10, 5, and 1% levels, respectively.

**Figure 3:**
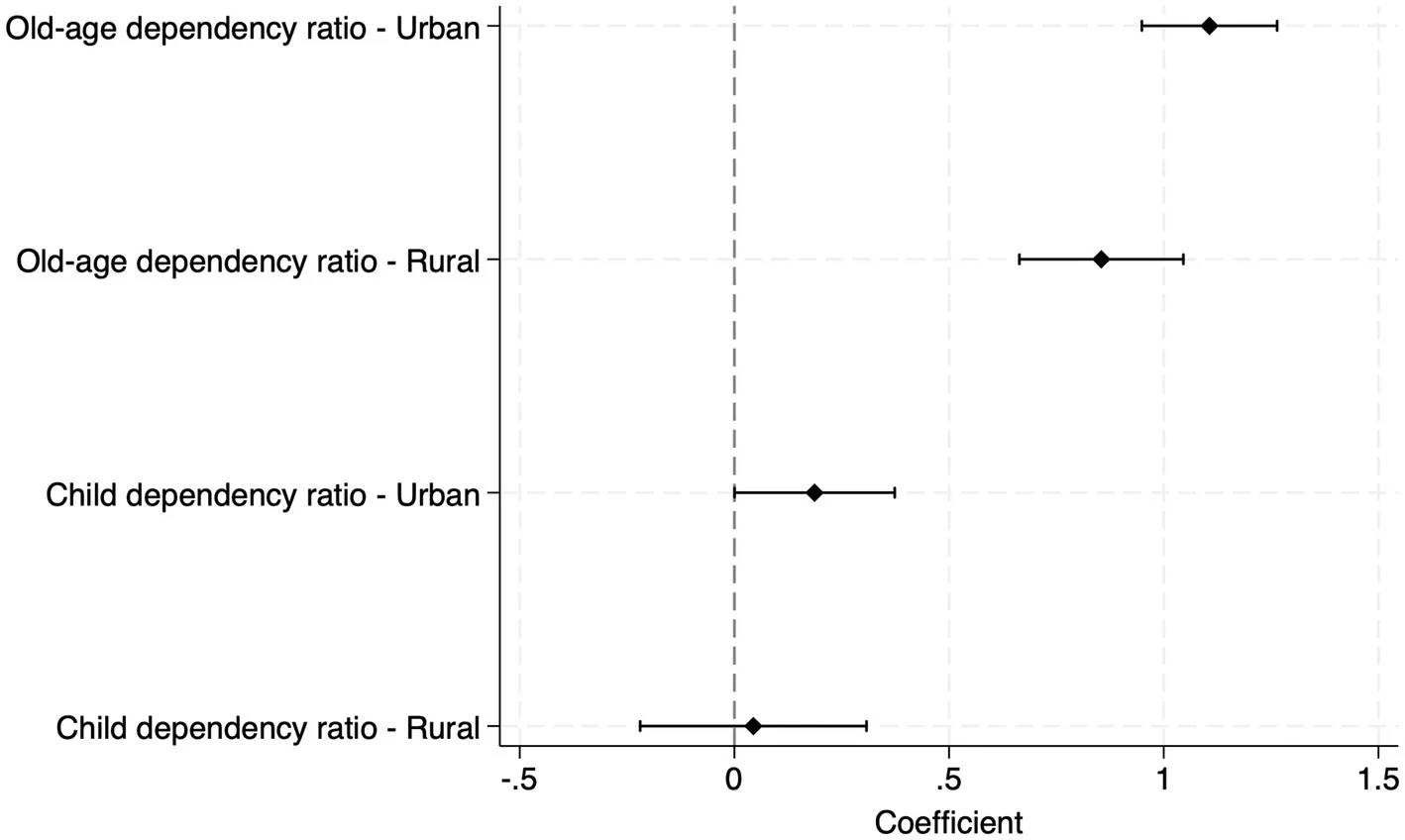
Urban–rural heterogeneity in broad expenditure coefficients. Points report subgroup coefficient estimates for the broad medical and healthcare expenditure specification, and horizontal lines denote 95% confidence intervals.

#### 3.5.1 Old-age dependency

Comparatively stable associations across groups. The old-age dependency ratio is positively and statistically significantly associated with broad medical and healthcare expenditure for both urban and rural households. The estimated coefficient is larger for urban households (1.1065) than for rural households (0.8547). A similar pattern emerges across income groups, with positive and statistically significant coefficients for both high-income and low-income households. These findings suggest that the association involving old-age dependency association is present across groups, although its magnitude varies.

#### 3.5.2 Child dependency

More heterogeneous and less stable associations. The child dependency ratio is positive and statistically significant for urban households (0.1868, *p* = 0.0496), but statistically insignificant for rural households (0.0442, *p* = 0.7426). Across income groups, the coefficient is positive but only marginally significant for high-income households (0.2294, *p* = 0.0817) and statistically insignificant for low-income households (0.0822, *p* = 0.3887). These results indicate that the child-dependency association is more sensitive to subgroup definition and is materially less stable than the association involving old-age dependency association.

The contrast suggests that broad healthcare demand may operate across households, whereas child-dependency spending associations depend more on resource flexibility, competing priorities, and context.

### 3.6 Supplementary instrumental-variable analysis

Given the non-random nature of household demographic composition, Table 5 reports a supplementary instrumental-variable diagnostic rather than a primary causal analysis.

**Table 5:** Supplementary instrumental-variable results.

Statistic	(1)	(2)	(3)	(4)	(5)
Model	Child first stage	Old-age first stage (prov.)	Old-age first stage (county)	Child 2SLS broad	Child 2SLS narrow
Key coefficient	0.0036 (0.0057)	−0.0200 (0.0354)	−0.0169 (0.0244)	−104.2501 (165.8094)	16.2257 (40.7687)
Approx. *F*-statistic	0.3976	0.3197	0.4793	0.3976	0.3976
Controls	Yes	Yes	Yes	Yes	Yes
Year fixed effects	Yes	Yes	Yes	Yes	Yes
*N*	47,749	47,664	47,664	47,749	35,482

Household-level clustered standard errors are reported in parentheses. All regressions control for household and household-head characteristics and include year fixed effects. *, **, and *** denote significance at the 10, 5, and 1% levels, respectively.

The child-dependency instrument interacts a post-2016 policy indicator with the number of married women of fertile age in the household; the instruments for old-age dependency are leave-one-out province-year and county-year pension coverage rates. Their use requires first-stage relevance and exclusion restrictions.

First-stage coefficients are statistically insignificant and F-statistics are far below conventional relevance thresholds. Correspondingly, second-stage estimates are imprecise and statistically insignificant, with large standard errors and unstable point estimates.

The IV exercise therefore does not provide credible causal identification. It is retained only as a diagnostic, and the main results remain descriptive and associational.

### 3.7 Descriptive complement: co-movement with other expenditure categories

Table 6 provides a descriptive complement by reporting weighted pooled cross-sectional correlations between narrow health/fitness expenditure and major household expenditure categories in the comparable 2017, 2019, and 2021 waves. The 2015 wave is excluded because its categories are not directly comparable. This available-case sample requires only narrow health/fitness expenditure, the relevant expenditure category, and survey weights, so it is larger than the complete-case baseline sample. The exercise provides context and does not alter the empirical design.

**Table 6:** Pooled cross-sectional co-movement between narrow health/fitness expenditure and other major household expenditure categories.

Measure	Weighted correlation	*N*
ln health/fitness exp. vs. ln total household consumption	0.252	95,662
ln health/fitness exp. vs. ln other household consumption	0.232	95,662
ln health/fitness exp. vs. ln food expenditure	0.159	95,658
ln health/fitness exp. vs. ln clothing expenditure	0.123	95,638
ln health/fitness exp. vs. ln residential expenditure	0.141	95,662
ln health/fitness exp. vs. ln household equipment expenditure	0.205	95,662
ln health/fitness exp. vs. ln transportation/travel expenditure	0.152	95,661
ln health/fitness exp. vs. ln education/culture expenditure	0.182	95,662
ln health/fitness exp. vs. ln medical/healthcare expenditure	0.231	95,662
ln health/fitness exp. vs. ln other expenditure	0.150	60,977

The table reports weighted pooled cross-sectional correlations based on the harmonized 2017, 2019, and 2021 CHFS waves. Household survey weights are used to preserve consistency with the CHFS sampling design and the weighted baseline analyses.

Narrow health/fitness expenditure is positively correlated with total consumption (0.252), consumption excluding health/fitness expenditure (0.232), and each reported category (0.123–0.231). Cross-sectionally, it therefore co-moves with broader spending capacity rather than showing an obvious displacement pattern.

## 4 Discussion

The findings reveal distinct associations of old-age and child dependency with different components of household health-related expenditure. First, the positive and comparatively stable association between the old-age dependency ratio and broad medical and healthcare expenditure is consistent with prior research linking population ageing to greater demand for healthcare and long-term-care services (2, 5, 8, 11). The parallel positive association with narrow health/fitness expenditure is broadly consistent with literature emphasizing active ageing, physical activity, and health-promoting behaviors among older adults (12–15). Relative to studies centered on necessity-driven medical spending, the present findings suggest that ageing may be associated with a broader household health-expenditure portfolio that includes preventive and health-promoting investment. This interpretation remains associational and should not be read as evidence that ageing causally increases either expenditure category.

Second, the weaker and less stable child-dependency results are consistent with two offsetting forces. Child dependency may strengthen household investment motives in children’s health, development, and organized activities (17, 18), while resource dilution and financial constraints may constrain discretionary sports and fitness expenditure (19–21). This interpretation also aligns with evidence that financial resources, parental time, and access conditions shape inequalities in physical activity and sports participation (21, 23–25). The coexistence of investment motives and resource constraints provides a plausible explanation for why the child-dependency estimates vary more across outcomes, samples, and household groups than the estimates for old-age dependency.

The mediation analysis provides evidence consistent with different potential pathways across the two dependency ratios. The statistically insignificant indirect effects through liquid assets do not support a detectable precautionary-saving pathway for old-age dependency in these data. For child dependency, the mediation results are consistent with the possibility that household disposable income and related resource constraints are relevant. These patterns are compatible with research on medical-expense risk, precautionary saving, and household financial resilience (6, 16, 22, 26) and on socioeconomic constraints in child health behavior and sports participation (21, 23–25). They do not establish that either pathway operates causally, because mediation estimates based on observational data may still reflect residual confounding.

Together, the findings show why demographic structure and health expenditure should be disaggregated: old-age and child dependency are associated with different expenditure components and plausible pathways. These are empirical benchmarks rather than causal estimates for future modeling and policy evaluation under demographic transition.

### 4.1 Public health implications

The findings have several implications for public health policy. First, the positive and stable association between old-age dependency and both expenditure outcomes suggests that population ageing is linked to a broader reallocation of household spending toward health-related categories. Policy responses should therefore not focus only on medical treatment or insurance coverage, but should also consider affordable preventive services, community fitness resources, and age-friendly opportunities for health maintenance.

Second, the child-dependency results are consistent with the possibility that household resource constraints matter for discretionary health-promoting expenditure. Policies that reduce the cost burden of raising children, improve community access to sports and physical-activity facilities, or support low-cost family health-promotion programs may have indirect benefits for preventive health behavior.

Because household contexts and dependency types differ, policy should distinguish households with discretionary room from rural or lower-income households that may need direct access support, and should tailor responses to healthcare demand-side pressures associated with older adults versus child-related resource constraints.

### 4.2 Limitations and future research

The study is limited by its observational design: the weak instruments preclude causal identification, and mediation estimates show only patterns consistent with potential pathways. Future work could exploit cohort variation, policy discontinuities, or other quasi-experimental settings.

The CHFS also lacks detailed measures of physical activity, facility access, time use, participation quality, and specific activity types. Richer data would better distinguish preventive investment from recreational spending.

The analysis focuses on static associations over a limited period. Dynamic, structural life-cycle, or overlapping-generations approaches could examine how old-age and child dependency operate over the life course and across generations.

Section 3.7 shows positive cross-sectional co-movement between health/fitness and other expenditure categories, but does not establish whether demographic change causes crowding out or complementarity. Richer expenditure detail and stronger identification are needed to study these allocation effects.

Finally, comparative work across countries or institutional settings would help assess external validity. Cross-country or cross-region studies could clarify how social protection systems, local service provision, and cultural norms shape the relationship between demographic structure and household expenditure.

## Data Availability

The China Household Finance Survey (CHFS) data analyzed in this study are available to researchers for academic purposes upon registration and application through the Survey and Research Center for China Household Finance at https://chfser.swufe.edu.cn/data/.
