# Supplementary material for: Demographic structure, consumption composition, and household health investment: evidence from sports and fitness expenditure
Source: Front Public Health. 2026 Sep 23;14:1815361. doi: 10.3389/fpubh.2026.1815361 (PMC13641993; doi:10.3389/fpubh.2026.1815361)
Supplement: Supplementary file 1 [file Table_1.DOCX]

**Supplementary Material**

**Supplementary Table S1. Descriptive statistics**

| **Variable** | **N** | **Mean** | **SD** | **Median** | **P25** | **P75** |
| --- | --- | --- | --- | --- | --- | --- |
| Broad medical and healthcare expenditure (ln+1) | 131398 | 6.8902 | 3.1792 | 7.7581 | 6.2166 | 8.9684 |
| Narrow health/fitness expenditure (ln+1) | 95662 | 0.9684 | 2.4454 | 0.0000 | 0.0000 | 0.0000 |
| Elderly dependency ratio | 112167 | 0.2052 | 0.4159 | 0.0000 | 0.0000 | 0.2500 |
| Child dependency ratio | 112167 | 0.2038 | 0.3183 | 0.0000 | 0.0000 | 0.3333 |
| Precautionary-saving proxy (ln+1) | 133970 | 7.6371 | 3.7444 | 8.5174 | 6.2166 | 10.3288 |
| Household disposable income per capita (ln+1) | 133970 | 9.1882 | 2.2130 | 9.6804 | 8.6613 | 10.4322 |
| Age of household head | 133926 | 55.1846 | 13.9072 | 55.0000 | 45.0000 | 65.0000 |
| Household head is male | 133969 | 0.7536 | 0.4309 | 1.0000 | 1.0000 | 1.0000 |
| Educational attainment of household head | 133769 | 3.4062 | 1.6630 | 3.0000 | 2.0000 | 4.0000 |
| Household head is married | 133853 | 0.8499 | 0.3571 | 1.0000 | 1.0000 | 1.0000 |
| Health status of household head | 133855 | 2.6548 | 0.9956 | 3.0000 | 2.0000 | 3.0000 |
| Average monthly working days of household head | 50546 | 23.6074 | 5.4510 | 24.0000 | 21.0000 | 28.0000 |
| Rural household | 133970 | 0.3385 | 0.4732 | 0.0000 | 0.0000 | 1.0000 |
| Household size | 133970 | 3.2382 | 1.5560 | 3.0000 | 2.0000 | 4.0000 |
| Total household consumption (ln+1) | 133970 | 10.7248 | 0.8764 | 10.7588 | 10.1659 | 11.2928 |
| Total household assets (ln+1) | 133970 | 12.6864 | 1.7203 | 12.8579 | 11.7146 | 13.8457 |

Note: Continuous variables are winsorized at the 1st and 99th percentiles. Expenditure variables are reported as ln(1+x).
